# Supplementary material for: Implementation of point-of-care ultrasound in the medical intensive care unit: A retrospective analysis of physician practices and patient outcomes
Source: PLoS One. 2025 Aug 28;20(8):e0330719. doi: 10.1371/journal.pone.0330719 (PMC12393712; doi:10.1371/journal.pone.0330719)
Supplement: S1 Table — (DOCX) [file pone.0330719.s001.docx]

## **Table S1:** **Summary statistics of MICU length of stay across physician POCUS implementation and confidence group** **(all data)**

| **Variable** | **level** | **N** | **Mean** | **Std** | **Minimum** | **Q1** | **Median** | **Q3** | **Maximum** | **P-value** |
| --- | --- | --- | --- | --- | --- | --- | --- | --- | --- | --- |
| Implementation | High | 320 | 7.32 | 6.99 | 1.00 | 3.00 | 5.00 | 9.00 | 47.00 | 0.8558 |
|  | Medium | 313 | 6.79 | 6.78 | 1.00 | 3.00 | 5.00 | 8.00 | 47.00 |  |
|  | Low | 203 | 7.06 | 7.18 | 1.00 | 3.00 | 5.00 | 8.00 | 47.00 |  |
| Confidence | High | 332 | 6.79 | 7.01 | 1.00 | 3.00 | 5.00 | 7.00 | 47.00 | 0.0751 |
|  | Medium | 254 | 6.74 | 6.57 | 1.00 | 3.00 | 5.00 | 8.00 | 47.00 |  |
|  | Low | 250 | 7.74 | 7.24 | 1.00 | 3.00 | 5.00 | 9.00 | 47.00 |  |
| Note: p-values based on Kruskal-Wallis test were reported. | | | | | | | | | | |
