# Supplementary material for: Implementation of point-of-care ultrasound in the medical intensive care unit: A retrospective analysis of physician practices and patient outcomes
Source: PLoS One. 2025 Aug 28;20(8):e0330719. doi: 10.1371/journal.pone.0330719 (PMC12393712; doi:10.1371/journal.pone.0330719)
Supplement: S2 Table — (DOCX) [file pone.0330719.s002.docx]

## **Table S2: Summary statistics of vent days across physician POCUS implementation and confidence group** **(ventilator positive data)**

| **Variable** | **level** | **N** | **Mean** | **Std** | **Minimum** | **Q1** | **Median** | **Q3** | **Maximum** | **P-value** |
| --- | --- | --- | --- | --- | --- | --- | --- | --- | --- | --- |
| Implementation | High | 140 | 3.49 | 2.73 | 1.00 | 2.00 | 2.00 | 5.00 | 15.00 | 0.7579 |
|  | Medium | 146 | 3.24 | 2.53 | 1.00 | 1.00 | 2.00 | 4.00 | 15.00 |  |
|  | Low | 97 | 3.52 | 2.94 | 1.00 | 1.00 | 3.00 | 5.00 | 13.00 |  |
| Confidence | High | 153 | 3.20 | 2.68 | 1.00 | 1.00 | 2.00 | 4.00 | 15.00 | 0.2496 |
|  | Medium | 116 | 3.45 | 2.80 | 1.00 | 1.00 | 3.00 | 4.00 | 15.00 |  |
|  | Low | 114 | 3.62 | 2.65 | 1.00 | 2.00 | 3.00 | 5.00 | 13.00 |  |
| Note: p-values based on Kruskal-Wallis test were reported. | | | | | | | | | | |
