# Supplementary material for: Implementation of point-of-care ultrasound in the medical intensive care unit: A retrospective analysis of physician practices and patient outcomes
Source: PLoS One. 2025 Aug 28;20(8):e0330719. doi: 10.1371/journal.pone.0330719 (PMC12393712; doi:10.1371/journal.pone.0330719)
Supplement: S3 Table — (DOCX) [file pone.0330719.s003.docx]

## **Table S3: Summary statistics of pressor days across physician POCUS implementation and confidence group** **(vasopressor positive data)**

| **Variable** | **level** | **N** | **Mean** | **Std** | **Minimum** | **Q1** | **Median** | **Q3** | **Maximum** | **P-value** |
| --- | --- | --- | --- | --- | --- | --- | --- | --- | --- | --- |
| Implementation | High | 84 | 2.36 | 1.71 | 1.00 | 1.00 | 2.00 | 3.00 | 7.00 | 0.6574 |
|  | Medium | 74 | 2.58 | 1.89 | 1.00 | 1.00 | 2.00 | 4.00 | 11.00 |  |
|  | Low | 61 | 2.44 | 1.92 | 1.00 | 1.00 | 2.00 | 3.00 | 11.00 |  |
| Confidence | High | 83 | 2.28 | 1.45 | 1.00 | 1.00 | 2.00 | 3.00 | 7.00 | 0.4333 |
|  | Medium | 63 | 2.76 | 2.20 | 1.00 | 1.00 | 2.00 | 4.00 | 11.00 |  |
|  | Low | 73 | 2.40 | 1.83 | 1.00 | 1.00 | 2.00 | 3.00 | 9.00 |  |
| Note: p-values based on Kruskal-Wallis test were reported. | | | | | | | | | | |
