# Supplementary material for: Implementation of point-of-care ultrasound in the medical intensive care unit: A retrospective analysis of physician practices and patient outcomes
Source: PLoS One. 2025 Aug 28;20(8):e0330719. doi: 10.1371/journal.pone.0330719 (PMC12393712; doi:10.1371/journal.pone.0330719)
Supplement: S1 Text — (DOCX) [file pone.0330719.s004.docx]

**Survey**

*Please write your name on the survey.*

*Thank you for agreeing to participate in this study.*

**For questions 1-4, please select the most applicable descriptor:**

1. Number of years in practice of Pulmonary Critical Care Medicine (attending level):
   1. <1
   2. 1-5
   3. 5-10
   4. 10-15
   5. >15
2. Your current number of years of bedside ultrasound use in your practice:
   1. <1
   2. 1-5
   3. 5-10
   4. 10-15
   5. >15
3. Your prior method of ultrasound training (please check only one; choose answer which applies best to your experience):
   1. Never trained and no experience
   2. Formal training course(s) alone, without practice opportunity
   3. Formal training course followed by supervised practice
   4. Formal training course followed by self-directed practice
   5. Supervised learning only
   6. Self-directed learning only
4. In our review of the MICU database from July 2016 to May 2017, the average number of patients in the MICU at any given time was 8 to 9. This is equivalent on average to 16 to 18 patient encounters per day. In your practice, of these 16 to 18 encounters, how many would you estimate included the use of bedside ultrasound?
   1. Write number:

| **Statement** | **1**  **Very**  **Ineffective** | **2**  **Moderately Ineffective** | **3**  **Neutral** | **4**  **Moderately Effective** | **5**  **Very Effective** |
| --- | --- | --- | --- | --- | --- |
| 5. Evaluation of pneumonia. |  |  |  |  |  |
| 6. Evaluation of pulmonary edema. |  |  |  |  |  |
| 7. Evaluation of pneumothorax. |  |  |  |  |  |
| 8. Evaluation of diaphragm function. |  |  |  |  |  |
| 9. Assessment of respiratory failure. |  |  |  |  |  |
| 10. Assessment of cardiac function. |  |  |  |  |  |
| 11. Assessment of IVC for preload responsiveness. |  |  |  |  |  |
| 12. For ruling out abdominal catastrophe or emergent surgical abdomen. |  |  |  |  |  |
| 13. Evaluation of intracranial pressure. |  |  |  |  |  |
| 14. Management of patients in cardiac arrest. |  |  |  |  |  |
| 15. Evaluation and management of shock. |  |  |  |  |  |
| 16. DVT study. |  |  |  |  |  |
| 17. Guidance for vascular access: central venous lines. |  |  |  |  |  |
| 18. Guidance for vascular access: peripheral venous lines. |  |  |  |  |  |
| 19. Guidance for vascular access: arterial lines. |  |  |  |  |  |
| 20. Endotracheal tube placement. |  |  |  |  |  |
| 21. To confirm gastric tube placement. |  |  |  |  |  |
| 22. Evaluation and management of pleural effusion. |  |  |  |  |  |
| 23. Evaluation of diaphragm function. |  |  |  |  |  |

**For questions 5-23 below, please indicate your level of agreement with regards to the effectiveness of bedside ultrasound in each situation by placing an “X” in the corresponding box:**

| **Statement** | **1**  **Very Misleading** | **2**  **Moderately Misleading** | **3**  **Not Misleading and Not Useful** | **4**  **Moderately Useful** | **5**  **Very Useful** |
| --- | --- | --- | --- | --- | --- |
| 24. Evaluation of pneumonia. |  |  |  |  |  |
| 25. Evaluation of pulmonary edema. |  |  |  |  |  |
| 26. Evaluation of pneumothorax. |  |  |  |  |  |
| 27. Evaluation of diaphragm function. |  |  |  |  |  |
| 28. Assessment of respiratory failure. |  |  |  |  |  |
| 29. Assessment of cardiac function. |  |  |  |  |  |
| 30. Assessment of IVC for preload responsiveness. |  |  |  |  |  |
| 31. For ruling out abdominal catastrophe or emergent surgical abdomen. |  |  |  |  |  |
| 32. Evaluation of intracranial pressure. |  |  |  |  |  |
| 33. Management of patients in cardiac arrest. |  |  |  |  |  |
| 34. Evaluation and management of shock. |  |  |  |  |  |
| 35. DVT study. |  |  |  |  |  |
| 36. Guidance for vascular access: central venous lines. |  |  |  |  |  |
| 37. Guidance for vascular access: peripheral venous lines. |  |  |  |  |  |
| 38. Guidance for vascular access: arterial lines. |  |  |  |  |  |
| 39. Endotracheal tube placement. |  |  |  |  |  |
| 40. To confirm gastric tube placement. |  |  |  |  |  |
| 41. Evaluation and management of pleural effusion. |  |  |  |  |  |
| 42. Evaluation of diaphragm function. |  |  |  |  |  |

**For questions 24-42 below, please provide your opinion regarding the utility of bedside ultrasound for the following uses in your practice by placing an “X” in the corresponding box:**

**Please provide additional comments regarding the use of ultrasound in MICU for the purposes discussed in this survey:**
